# Healthcare resource utilization and costs associated with anogenital warts in Morocco

**DOI:** 10.1186/s13027-021-00403-1

**Published:** 2021-11-14

**Authors:** Myriam Berrada, Ryan Holl, Tidiane Ndao, Goran Benčina, Siham Dikhaye, Abdelilah Melhouf, Soumiya Chiheb, Khalid Guelzim

**Affiliations:** 1 MSD, Casablanca, Morocco; 2 MSD, Kriens, Switzerland; 3 Center for Observational and Real-World Evidence, MSD, Madrid, Spain; 4 Department of Dermatology, Mohammed VI University Hospital of Oujda, Oujda, Morocco; 5 Laboratory of Epidemiology, Clinical Research and Public Health, Faculty of Medicine and Pharmacy, Mohammed the First University of Oujda, Oujda, Morocco; 6 Department of Gynecology – Obstetrics, Faculty of Medicine and Pharmacy Hassan II, Sidi Mohamed Ben Abdellah University, Fez, Morocco; 7 Department of Dermatology, University Hospital Ibn Rochd of Casablanca, Casablanca, Morocco; 8 Department of Gynecology and Obstetrics, Military and Training Hospital Mohammed V, Rabat, Morocco

**Keywords:** Anogenital warts, Genital warts, Condyloma acuminatum, Papillomavirus infections, Epidemiology, North Africa, Health care utilization, Health care costs

## Abstract

**Background:**

Human papillomavirus (HPV), primarily genotypes 6 and 11, cause the majority of cases of anogenital warts (AGW). Although benign, AGW are associated with a substantial economic and psychosocial burden. Several vaccines have been developed to prevent HPV. The objective of this study was to describe the epidemiology and healthcare resource utilization of AGW in Morocco, as well as the associated costs of treatment from the public healthcare perspective.

**Methods:**

This was a descriptive analysis of questionnaire data obtained via a Delphi panel. The panel consisted of 9 physicians practicing in public hospitals in Morocco (4 dermatologists and 5 obstetricians/gynecologists). The questionnaire collected data on physician and practice characteristics, diagnostic tests and procedures, treatments, and follow-up (including recurrence) of patients with AGW. Questionnaire items on which ≥ 70% of respondents agreed were considered as having consensus. Costs associated with diagnosis, treatment, and follow-up were calculated in Moroccan dirham (MAD) and converted to euros (€) based on official national price lists for public hospitals and the HCRU estimates from the questionnaire.

**Results:**

The physician-estimated prevalence of AGW in Morocco was 1.6%-2.6% in women and 2.0%-5.3% in men. A mean (median) of 6.4 (4) patients per month per physician sought medical attention for AGW. Simple observation was the most common diagnostic method for AGW in both men and women, and excision was the most prescribed therapy (75%), requiring a mean of 2 visits. Recurrence occurred in approximately 27% of patients. The cost per case of managing AGW, including recurrence, was estimated at 2182–2872 MAD (€207–272) for women and 2170–2450 MAD (€206–233) for men. The total annual cost of medical consultations for AGW in Morocco ranged from 3,271,877 MAD to 4,253,703 MAD (€310,828–404,102).

**Conclusions:**

Expert consensus indicates that AGW represent a significant burden to the Moroccan public healthcare system. These data can inform policy makers regarding this vaccine-preventable disease.

**Supplementary Information:**

The online version contains supplementary material available at 10.1186/s13027-021-00403-1.

## Background

Human papillomavirus (HPV) is well known as a causative agent of anogenital cancers [1], and several vaccines have been developed to prevent cancers associated with multiple oncogenic HPV genotypes [2]. However, HPV also causes the majority of cases of anogenital warts (AGW) [1], which are benign proliferative lesions found on the epithelium of the genitalia, anus, or perianal area. AGWs, also called condyloma acuminata, are among the most common sexually transmitted viral infection in the world for both men and women. More than 90% of cases of AGWs are caused by low-risk HPV types 6 or 11 while 10% of cases are associated with high-risk HPVs [3]. Furthermore, HPV appears to play a strong role in the development of anogenital cancers and high-risk HPV types 16 and 18 can be detected in more than 70% of cervical cancers [4, 5]. Although benign, AGW are associated with a substantial economic and psychosocial burden [6, 7]. Both the quadrivalent and the nonavalent HPV vaccines target HPV 6 and 11, providing protection against the cause of 90% of AGW [2].

Based on a systematic review of studies from Europe, the Americas, East Asia, and Australia, AGW are present in 0.2% to 5.0% of the general population (both males and females), but epidemiological data from Africa are limited [8]. A review of studies from sub-Saharan Africa found only two reports of the prevalence of AGW through the year 2012 [9]. AGW were present in 1.9% of men and 0.4% of women attending a sexually transmitted infection clinic in Harare, Zimbabwe, in 1980 [10]; and among female sex workers in Burkina Faso in 2003–2006, AGW were present in 1.6% of HIV-negative women and 7.0% of HIV-positive women [11]. More recent studies have reported prevalence values for AGW of 1% in HIV-negative women in Nigeria [12] and 2% in women of reproductive age in Swaziland [13]. However, epidemiological data from Northern Africa are lacking.

Morocco is one of the few African countries to have plans for an HPV vaccination program and to offer the vaccine in the private sector [14, 15]. The high rates of cervical cancer and cervical cancer-related mortality in Morocco are well documented [16], but less is known about the prevalence and burden of AGW in this North African country. The main objective of this study was to characterize the epidemiology and healthcare resource utilization of patients with AGW in Morocco and then calculate the associated costs of treatment from the public healthcare perspective. This information will provide a basis for evaluating the benefits of HPV vaccination beyond cancer prevention.

## Methods

### Study design

This was a descriptive study utilizing a Delphi panel as a means to build consensus on the patterns of management of AGW in Morocco. The Delphi technique synthesizes the opinions of a panel of experts via multiple rounds of questionnaires. In the current study, two rounds of individual and anonymous questionnaires were administered to each panel member in December of 2019, and consensus was defined as agreement among  ≥ 70% of the panelists [17]. After round 1, a report was written to summarize areas of consensus and non-consensus, and a second questionnaire was administered to further query the areas of non-consensus. In this second round, panel members were asked to review the outcome of the first round and either agree with the reported outcome or recommend and justify changes. Round 2 was followed by a group discussion (via teleconference or e-mail) in order to reach consensus on any outstanding questionnaire items. For patterns of diagnosis, treatment, and follow-up that attained consensus, the associated costs were calculated. The study protocol was approved by a national ethics committee, the Comité D’Ethique pour la Recherche Biomédicale (CERB).

### Study sample

Ten experts in the field of AGW were recruited, specifically dermatologists and obstetrician/gynecologists. In order to be considered for participation in the panel, subject matter experts had to (1) be based in Morocco and known as a subject matter expert in the management of AGW, (2) have an active medical practice where patients with AGW are treated, and (3) work at least part-time in the public sector. A signed confidentiality disclosure agreement was required for participation. Individuals were excluded from participation if they (1) might be involved in the decision to include HPV vaccination in the National Immunization Program, (2) only worked in private medical offices and/or private clinics, or (3) failed to sign the confidentiality disclosure agreement for the study.

### Study questionnaire

The full questionnaire from round 1 is shown in the Appendix. It collected information in 4 main sections: (1) information about the panelist (e.g., sex, age, institution, medical field, years in practice); (2) clinical practice characteristics (number of patients seen per month, gender and age of patients, prevalence of AGW); (3) institution-level data on diagnosis and treatment of AGW, availability of guidelines, and healthcare resource utilization (HCRU; i.e., hospitalization); and (4) outcomes of AGW management (side effects, complications, referrals to other specialties, follow-up, and recurrences). Questions generally took one of two forms, asking either for a number (e.g., percentage of patients, duration of treatment in years, estimate of prevalence, etc.) or for a categorical designation (e.g., yes/no; or never, sometimes, regularly, or always).

### Statistical analysis

One panelist cancelled his participation before the first questionnaire was administered, leaving a total study sample of 9. All questionnaires of the panelists who met the inclusion and exclusion criteria and who agreed to participate in the Delphi panel were analyzed and constitute the full analysis data set. The analysis of each round of questionnaires was performed after the last panelist had completed the scheduled assessments and the database had been cleaned and locked. All analyses were descriptive and were performed by a third-party vendor using SAS® software (SAS Institute, North Carolina, USA).

Responses measured as continuous variables were summarized by the number of missing and non-missing observations, as well as the mean, standard deviation, median, minimum, interquartile range (IQR), and maximum values. Due to the small sample size, the results for continuous variables are generally reported in this paper as the median and IQR, with exceptions indicated in the tables or text. Categorical variables were described by the number and the percentage of answers in each category, as well as the number of missing data points, if any; the percentages are generally presented in the text. Percentages are based on non-missing observations; panelists had the freedom to answer or not answer specific questions, and the missing data were not imputed. For categorical data, consensus was defined by agreement of ≥ 70% of the panelists on an item or when the number of respondents answering (always + regularly) or (never + sometimes) was ≥ 70%.

For questions related to diagnosis, treatment, follow-up, and recurrence, costs were calculated in Moroccan dirham (MAD) for items reaching consensus. The base cost of each diagnostic method and of treatment, follow-up, and recurrence was obtained from the official national nomenclature lists [18–20] as applied to public healthcare settings. The conversion of base costs to total costs is explained in Additional file 1: Table S1. In brief, the total cost was the sum of (1) the cost of all diagnostic tests given regularly or always, (2) the final cost of the diagnostic method, (3) the cost of first-line treatment, and (4) the cost of follow-up via simple observation. For cost analyses, recurrence was defined as another round of treatment and follow-up. Costs are presented in MAD and in euros (€) using the exchange rate of 1 MAD = 0.095 €.

The annual national cost of AGW management was also calculated based on the estimated annual number of patients and unit costs for healthcare resources. Taking into account the main Moroccan public hospitals with dermatological and gynecological departments where patients are treated for AGW (26 departments including the 9 departments represented by panelists), and using the panelists’ estimate of a mean of 6.4 patients seen per month, this represents 1997 medical consultations for AGW per year. The minimum and maximum costs per consultation, adjusted by the cost of recurrence at a frequency of 27%, were calculated separately for men and women and then multiplied by 1997 to obtain the total, population level cost for men and women. These values were then summed via a weighted average (27% men, 73% women) to give the total overall estimated cost of management of AGW in the Moroccan public healthcare sector.

## Results

### Physician and practice characteristics

Nine physicians participated in the Delphi panel (Table 1). Six were males, 5 were obstetricians/gynecologists, and the median age was 50 years. The median practice duration was 20 years, and panelists reported seeing a median of 4 patients with AGW per month (mean 6.4). Among patients with AGW, panelists estimated that 73% were female, 20% were under age 20, and 79% were presenting for the first time. The estimated prevalence of AGW in Morocco was 1.6%-2.6% in women and 2.0%-5.3% in men.

**Table 1:** Physician and practice characteristics

	N = 9
Physician characteristics	
Age, median (range) years	50 (43–57)
Sex, n (%)	
Male	6 (67%)
Female	3 (33%)
Specialty, n (%)	
Gynecology/obstetrics	5 (56%)
Dermatology	4 (44%)
Years in specialty practice, median (IQR)	20 (13–24)
Practice characteristics	
Patients seen per month, median (IQR)	240 (200–250)
AGW patients seen per month, median (IQR)^A^	4 (2–5)
Duration of care for patients with AGW, median (IQR) years	20 (17–20)
Distribution of AGW patients, median (IQR) percentage^B^	
Male	37 (30–70)
Female	70 (30–99)
Aged < 20 years	20 (14–25)
First presentation	79 (50–80)

AGW, anogenital warts; IQR, interquartile range

^A^The mean value was 6.4, which was the number used in the calculation of annual national costs

^B^The distribution of male and female patients was re-queried in round 2, with a result of 73% female and 27% male. This is the result reported in the text

### Diagnosis of AGW

Departments treating patients with AGW most commonly included gynecology, obstetrics, and dermatology. Diagnostic tests given always or regularly to patients presenting with AGW were HBV serology, HCV serology, VDRL for syphilis, and ELISA for HIV (Table 2). Cervical cytology was reported as being used always or regularly by 6 out of 9 panelists (67%). When asked to rank the procedures used to diagnose AGW, physicians ranked simple observation, vulvar colposcopy, and biopsy as first, second, and third, respectively, for women, while simple observation was universally used for men (Table 3). Women often required more than one visit to complete the diagnosis, whereas men usually required just one visit (Table 3). All panelists reported that a medical consultation is systematically conducted in the sexual partners of patients with AGW, with the same diagnostic procedures. Seven of the 9 panelists said that laboratory tests were systematically requested for the sexual partners.

**Table 2:** Diagnostic tests given to patients with anogenital warts^A^

Test	Never or sometimes	Regularly or always
HBV serology	–	100%
HCV serology	–	100%
VDRL test for syphilis	–	100%
ELISA for HIV	–	89%
Biopsy	89%	–
PCR for HPV	89%	–
Cervical cytology	33%	67%
Penescopy	100%	–
Anoscopy	100%	–
Other	100%	–

HBV, hepatitis B virus; HCV, hepatitis C virus; VDRL, Venereal Disease Research Laboratory; ELISA, enzyme-linked immunosorbent assay; HIV, human immunodeficiency virus; PCR, polymerase chain reaction; HPV, human papillomavirus

^A^The percentage of physicians with the indicated answers is shown

**Table 3:** Diagnosis of anogenital warts

Test	Rank	Average number of visits
Women		
Simple observation	1	1.2
Vulvar colposcopy	2	1.6
Biopsy	3	1.8
Men		
Simple observation	1	1
Biopsy^A^	-	-

^A^Eight of the 9 physicians reported not using biopsy to diagnose men with anogenital warts

### Availability of resources

Eight of the 9 panelists reported that they have no available clinical practice guidelines for the treatment of AGW at their institution, and for all 9, the main missing resource at their institution was pharmacological treatments.

### Treatment and follow-up

Ablative therapy was reported by 8 of the 9 panelists as the primary first-line treatment. The primary reason for this was the lack of availability of pharmaceutical treatments. Among ablative treatments, excision was ranked #1 by 6 of 8 respondents, and for most panelists (75%), there was no alternative treatment (other choices were laser and cryotherapy). Excision required an estimated mean of 2 visits. The treatment choice was guided firstly by the number, size, and morphology of lesions; secondly by the cost; and thirdly by patient preference. When asked which kind of therapy is considered as the second line of treatment, excision was cited always or regularly by 5 of the 6 respondents (83%). Among patients receiving ablative treatments, panelists reported that side effects arose sometimes (67%) or regularly (33%). Side effects included crust formation, burning, itching, and swelling. Only one panelist reported treating patients with pharmacological treatments and reported that the patients receiving pharmacological treatment sometimes had side effects, including itching, burning or swelling, and crust formation. All panelists reported that complications arose never or sometimes. Seven of the 9 panelists (78%) declared that their patients with AGW were never hospitalized for more than 24 h.

The consensus (89%) was that 3 follow-up consultations were required per patient per year and that approximately 27% of patients treated for AGW had a recurrence. Recurrence was treated preferentially with excision (67% always or regularly). Patients with recurrence were rarely referred to other specialties (100% never or sometimes).

### Cost of AGW

The estimated total costs of a case of AGW in women and men are shown in Table 4. The cost estimates for women differed according to the diagnostic procedure used. The estimated cost of a case of AGW diagnosed by simple observation was 1442–1610 MAD (€137–153) in women and 1430–1590 MAD (€136–151) in men. The cost was lower in men than women because of the lower estimated number of visits required to obtain the diagnosis. Recurrence added 740–860 MAD (€70–82) to the expected costs in each case, giving totals of 2182–2470 MAD (€207–235) in women and 2170–2450 MAD (€206–233) in men.

**Table 4:** Estimated total costs

	Total cost without recurrence		Total cost with recurrence	
	MAD	Euros	MAD	Euros
Women diagnosed by…				
Simple observation	1442–1610	136.99–155.90	2182–2470	207.29–237.60
Vulvar colposcopy	1530–1650	145.35–156.75	2270–2510	215.65–238.45
Biopsy	1892–2012	179.74–191.14	2632–2872	250.04–272.84
Men diagnosed by simple observation	1430–1590	135.85–151.05	2170–2450	206.15–232.75

Total cost without recurrence is the sum of (1) the cost of all diagnostic tests given regularly or always, (2) the final cost of the diagnostic method, (3) the cost of first-line treatment, and (4) the cost of follow-up via simple observation (see Supplementary Table S1). Simple observation had a range of costs, resulting in a range of estimated total costs. For total cost with recurrence, the cost of another round of treatment and follow-up was added to the initial cost estimate

MAD, Moroccan dirham

Based on the mean number of patient consultations reported by the panelists in this study (6.4 per month), the existence of 26 departments in Morocco (9 included in this study), and a 27% rate of recurrence, the total annual cost of medical consultations for AGW in Morocco ranged from 3,271,877 MAD to 4,253,703 MAD (€310,828–404,102).

## Discussion

This study reports the methods of diagnosis and treatment of patients with AGW in Morocco, based on the consensus achieved among a panel of expert physicians. The panelists provided estimates of the monthly number of consultations for AGW and the number of visits needed to diagnose AGW. These data were used to calculate the costs associated with management of AGW in Morocco, both per case and nationally.

A systematic review by Raymakers et al*.* summarized what is currently known about the economic burden of AGW [6]. Included studies were from the United States, Europe, and Australia, as no published information was found in other regions. One publication from Spain is relevant here, based on geographical proximity to Morocco. Castellsague et al*.* used medical records from 2005 and several national cost databases to determine the HCRU and costs associated with AGW in Spain [21]. In this study, the mean number of physician visits for an episode of AGW in 2005 was 3.8. Approximately 75% of patients were prescribed medication for AGW at least once. The adjusted mean cost per patient was €833 from the Spanish National Health Service perspective, and the national annual cost was €47 million. These numbers are all quite different from those found in the current study: 6–7 visits per case (2 more for recurrence), rare to no use of prescription medication, and costs of €206–273 per patient and €310,828–404,102 nationally in Morocco.

Although differences in study designs and populations make it difficult to directly compare estimated costs, some general observations of Raymakers et al*.* [6] may lend insight into the findings of the current study. The first was that, in almost all previous studies on the cost of AGW, including Castellsague et al. [21], costs were higher in women than in men, a finding with which our results agreed. Secondly, the main cost driver for AGW was physician visits, and a majority of previous studies reported that women had more visits than men, a trend consistent with our findings, and one that explains why costs are higher in women. Finally, in countries where both cervical cancer and AGW cost data are available (the United States and the United Kingdom), the national cost of AGW represents a significant proportion of the national cost of cervical cancer, suggesting that evaluations of the cost effectiveness of quadrivalent and nonavalent HPV vaccination should take not only cervical cancer but also AGW (and other HPV-related diseases) into account.

Most previous studies of HPV-related disease in Morocco have focused on cervical cancer [22–24]. Although according to GLOBOCAN 2018 data, Northern Africa has a low incidence of cervical cancer relative to other world regions [25], Morocco has the highest incidence and mortality rates among North African countries [26–28]. As of 2018, the age standardized incidence of cervical cancer is 17.2 per 100,000 women per year in Morocco (compared to 7.2 in Northern Africa and 13.1 worldwide), and the age-standardized mortality rate is 12.6 per 100,000 women per year (compared to 5.1 in Northern Africa and 6.9 worldwide) [26]. In part because of these regionally high rates, an economic analysis of countries in the extended Middle East and North Africa found that HPV vaccination would be cost effective in Morocco, up to a cost of $100 (international dollars) per girl, in terms of cervical cancer cases avoided [29].

While cervical cancer is the primary focus of Morocco’s planned HPV vaccination program, the prevention of AGW using the quadrivalent or nonavalent vaccine will provide additional benefits. In order for the full benefit of the HPV vaccination program to be realized, information on the epidemiological burden and cost of AGW is needed. This study is the first to provide such data.

The primary limitation of this study is that the data are based on expert opinion rather than objective and quantitative medical records or administrative databases. However, we note that previous studies of the HCRU and cost associated with AGW in other countries have used expert opinion (i.e., physician surveys) as a data source [21, 30–32]. Furthermore, the study includes a total of 9 physicians (4 dermatologists and 5 obstetricians/gynecologists). It is possible that the findings of the present study would have differed if physicians of other specialties were included. However, based on our investigations, most patients are referred to either a dermatologist or obstetrician so it is unlikely that including physicians of other specialties would change the outcomes of the study. Also, this analysis reflects patients treated only in the public sector, which almost certainly resulted in an underestimation of costs at the national level. The choice of ablative treatments to resolve AGW was determined for most of the panelists by the unavailability of pharmacological treatments. This is a limitation not only of this study, but of the Moroccan healthcare system, which is a resource-limited setting. For the cost of recurrence, it was assumed that the treatment and the number of follow-up visits were the same as for primary AGW, which may not always be the case. In previous studies, costs of recurrent or resistant cases were higher than for incident cases [6, 21]. Likewise, national cost estimates assumed that physicians outside our panel used the same diagnosis and treatment strategies as our panel. Finally, this study did not estimate the indirect costs associated with days absent from work or disability caused by treatment of AGW. A lack of information on the indirect costs and humanistic burden of AGW characterizes the field as a whole [6] and should be the subject of future studies worldwide.

## Conclusions

This study used expert consensus on diagnosis and treatment practices to demonstrate that AGW represent a burden to the Moroccan public healthcare system. These data are the first available on the management of AGW in Morocco and can inform policy decisions regarding this vaccine-preventable disease.

### Supplementary Information

**Additional file 1**. **Table S1.** Base costs for diagnosis, first-line treatment, follow-up, and recurrence^A^.

## Data Availability

All data generated or analyzed during this study are included in this published article and its supplementary information files.

## Appendix: Questionnaire, Round 1

**Table:**

Section	Question	
II	1	In average, to how many do you offer care per month?
	1	In average, to how many patients with anogenital warts do you offer care per month?
	2	Time offering care to patients with anogenital warts: (years)
	3	From your patients with anogenital warts, what percentage are: (a) male, (b) female?
	4	From your patients with anogenital warts, what percentage is younger than 20 years of age?
	5	From your patients with anogenital warts, what percentage presents the condition for the first time?
	6	From your patients with anogenital warts for the first time, what percentage are: (a) male, (b) female
	7	From your patients with anogenital warts for the first time, what percentage is younger than 20 years of age?
	8	In the United States, the prevalence of anogenital warts in men ranges between 2 and 11%, while in women, ranges between 1 and 3%. What do you consider to be the prevalence of anogenital warts in your institution?
IIIA	9	At your institution, what department(s) offers care to patients with anogenital warts?
	10	In addition to the clinical review, how often does your institution conduct diagnosis tests to patients with anogenital warts? (never, sometimes, regularly, always)
	11	How often the following diagnosis tests are ordered for patients with suspected anogenital warts at your institution? HBV serology, HCV serology, ELISA for HIV, VDRL for syphilis, biopsy, PCR for HPV, cervical cytology, penescopy, anoscopy, other (never, sometimes, regularly, always)
	12A	In women with anogenital warts, what are the diagnosis procedures used at your institution and the average number of visits for each procedure? (please classify from the most commonly used to the least) Vulvar colposcopy, biopsy, simple observation, other (specify) (rank of use [1 for the most commonly used], average number of visits)
	12B	In men with anogenital warts, what are the diagnosis procedures used at your institution and the average number of visits for each procedure? (please classify from the most commonly used to the least) Biopsy, simple observation, other (specify) (rank of use [1 for the most commonly used], average number of visits)
	13A	At your institution, is a revision systematically conducted in sexual partners of patients with anogenital warts? (yes, no) [if “NO” go to question 15A]
	13B	If “YES”, what are the diagnosis procedures conducted and the average number of visits for each procedure? (please classify from the most commonly used to the least) Vulvar colposcopy, biopsy, simple observation, other (specify) (rank of use [1 for the most commonly used], average number of visits)
	14A	At your institution, are laboratory tests systematically requested from sexual partners of patients with anogenital warts? (yes, no) [if “NO” go to question 16]
	14B	If “YES”, what one(s) of the following are the most commonly requested? (please choose the 3 most commonly requested and rank from 1 (most requested) to 3 (less requested)) HBV serology, HCV serology, ELISA for HIV, VDRL for syphilis, biopsy, PCR for HPV, cervical cytology, penescopy, anoscopy, other
IIIB	15	At your institution, are there available clinical practice guidelines for the treatment of anogenital warts? (yes, no) [if “NO” go to question 18]
	16A	Do you have all needed resources for the treatment of anogenital warts as per the guidelines? (yes, no) [if “YES” go to question 17]
	16B	If the answer is “no”, what resources are lacking?
IIIC	17	At your institution, how often patients with anogenital warts receive specific type of treatment? (never sometimes, regularly, always)
	18A	At your institution, which kind of therapy is considered as the first line of treatment for anogenital warts? Pharmacological treatments, ablative treatments
	18B	Why?
	18C	For either Pharmacological or Ablative, which treatment is the most commonly prescribed as the first line of treatment at your institution, and the average number of visits for each? (please classify from the most commonly prescribed to the least) Pharmacological: Trichloroacetic acid, Podophyllin, Imiquimod, Other. Ablative: Cryotherapy, Excision, Laser surgery, other. [Rank (1 for the most commonly prescribed). Average number of visits]
	18D	For which reason(s) this choice is made at your institution? (please choose the 3 principal reasons and rank from 1 (most important) to 3 (less important)) Number, size, site and morphology of lesions, convenience, patient preference, adverse effects, treatment cost, provider experience
	19	At your institution, which kind of therapy is considered as the second line of treatment for anogenital warts? Podophyllin, imiquimod, cryotherapy, trichloroacetic acid, excision, laser surgery, other. (never, sometimes, regularly, always)
	20A	At your institution, how often do patients with anogenital warts get hospitalized more than 24 h for treatment? (never sometimes, regularly, always)
	20B	In average how many days do patients with anogenital warts remain in the hospital? (days)
IV	21A	How often do patients treated for anogenital warts have side effects? (never sometimes, regularly, always)
	21B	Do patients treated for anogenital warts have the following side effects? Burning, itching, or swelling, crust formation, discoloration, others (never, sometimes, regularly, always)
	22A	Do patients treated for anogenital warts have complications? (never sometimes, regularly, always)
	22B	How often do patients treated for anogenital warts have the following complications? bleeding, infection, deformity, other (never, sometimes, regularly, always)
	23A	At your institution, how often patients with anogenital warts are referred to other specialties? (never sometimes, regularly, always)
	23B	Mention which specialties: surgery, gynecology, urology, infectiology, dermatology, psychology, psychiatry, otorhinolaryngology, other (never, sometimes, regularly, always)
	24	In average, how many follow-up consultations are given to patients with anogenital warts each year?
	25A	At your institution, how many patients treated for anogenital warts have recurrences
	25B	At your institution, are photos taken from patients with recurrences? (never, sometimes, regularly, always)
	25C	At your institution, what therapies are considered for the treatment of patients with anogenital warts when they recur? podophyllin, imiquimod, cryotherapy, trichloroacetic acid, excision, laser surgery, other (never, sometimes, regularly, always)
	26A	At your institution, are patients who recur after treatment for anogenital warts referred to other specialties? (yes, no)
	26B	To which of the following specialties are patients with recurrences from anogenital warts referred to? Surgery, gynecology, urology, infectiology, dermatology, psychology, psychiatry, otorhinolaryngology, other (never, sometimes, regularly, always)

Section I of the questionnaire asked about physician characteristics.

## Abbreviations

AGW: Anogenital warts
ELISA: Enzyme-linked immunosorbent assay
HBV: Hepatitis B virus
HCRU: Healthcare resource utilization
HCV: Hepatitis C virus
HIV: Human immunodeficiency virus
HPV: Human papillomavirus
IQR: Interquartile range
MAD: Moroccan dirham
PCR: Polymerase chain reaction
VDRL: Venereal Disease Research Laboratory
