# Supplementary material for: Healthcare resource utilization and costs associated with anogenital warts in Morocco
Source: Infect Agent Cancer. 2021 Nov 14;16:64. doi: 10.1186/s13027-021-00403-1 (PMC8591871; doi:10.1186/s13027-021-00403-1)
Supplement: Supplementary file 1 — Additional file 1. Table S1. Base costs for diagnosis, first-line treatment, follow-up, and recurrenceA. [file 13027_2021_403_MOESM1_ESM.docx]

## Supplementary Material

Table S1. Base costs for diagnosis, first-line treatment, follow-up, and recurrence ^A^

|  |  |  | Final cost | |
| --- | --- | --- | --- | --- |
|  | Base cost (MAD) | Multiplier | MAD | Euros |
| Diagnosis |  |  |  |  |
| Tests given regularly or always |  |  |  |  |
| HBV serology | - | - | 108 | 10.26 |
| HCV serology | - | - | 270 | 25.65 |
| VDRL for syphilis | - | - | 72 | 6.84 |
| ELISA for HIV | - | - | 180 | 17.10 |
| Diagnostic method |  |  |  |  |
| Simple observation | 60-100 | 1.2 (women)  1 (men) | 72-120 (women)  60-100 (men) | 6.84-11.40 (women)  5.7-9.5 (men) |
| Vulvar colposcopy | 100 | 1.6 | 160 | 15.20 |
| Biopsy | 290 | 1.8 | 522 | 49.59 |
| First-line treatment (excision) | 280 | 2 | 560 | 53.20 |
| Follow-up (simple observation) | 60-100 | 3 | 180-300 | 17.10-28.50 |
| Recurrence treatment (excision) | 280 | 2 | 560 | 53.20 |

ELISA, enzyme-linked immunosorbent assay; HBV, hepatitis B virus; HCV, hepatitis C virus; HIV, human immunodeficiency virus; MAD, Moroccan dirham; VDRL, Venereal Disease Research Laboratory

^A^ Base costs are per visit, and the multiplier is the average number of visits.
